# Brain-based learning in design and visual arts education: a bibliometric assessment of Scopus indexed literature

**DOI:** 10.12688/f1000research.110294.1

**Published:** 2022-04-08

**Authors:** Hala A. El-Wakeel, Reham Abdellatif, Dalia Hussain Eldardiry, Deema F. Al-Saleh, Mai I. Shukri, Khadeeja M N Ansari

**Affiliations:** 1 Interior Design Department, College of Design, Imam Abdulrahman Bin Faisal University, Dammam, Eastern Province, PO 1980, Saudi Arabia

**Keywords:** Brain-based learning; Design Education; Creativity; Visual Arts; Bibliometric assessment; Scopus indexed journals; Scientometric analysis.

## Abstract

Background: This study aims to critically review, quantify, and assess research outcomes on brain-based learning with an evidence-based study on Scopus indexed literature, with a focus to understand the evolution structure and growth, detect trends, subject development, and most importantly, identify the gaps in the published body of literature that relates brain-based learning to design and visual arts education.

Methods: Various scientometric tools were used to map, visualize, and analyze 186 research publications, indexed in Scopus in a twenty-year timespan ‘2001-2021’. Annual publication trends, relevant sources, prolific authors, authorship patterns, productive organizations and countries, funding agencies, keyword co-occurrence analysis, and thematic evolution mapping on brain-based learning publications were examined in this study.

Results: Despite the significance to apply brain-based learning strategies in design and visual arts education to boost students’ knowledge and creative skills, the findings show a decline in quantities and growth patterns in brain-based learning research directed towards design disciplines in the past twenty years. Among the identified (186) documents published in (128) sources, with (1013) citations, the study detected only (57) research (30%) that were related to ‘design education,’ including those focusing on ‘instructional design, ‘and ‘syllabus design’ whereas only (3) articles were in ‘design and visual arts’ disciplines.

Conclusion: These rather small numbers reflect the big gap in the current body of literature that associates brain-based learning with creativity-based disciplines, specifically in design and visual arts education. This infers the necessity to direct the attention of academics, researchers, and educationalists in the fields of design and arts towards brain-based learning applications, research and pedagogy.

## Introduction

### Brain-based learning definition and significance

Brain-based learning (BBL) is perceived as a core theory; it represents a learning paradigm that takes a holistic approach, looking at teaching and learning developmentally, socio-culturally, and in other broader ways ( Caine & Caine, 1995). It involves accepting the rules of the human brain processes and functions and then designing instruction, accordingly, achieving meaningful learning ( Duman, 2010). BBL includes specific learning processes based on how pupils are motivated, how attention works, how memories are created, how conceptual knowledge is acquired, how information is presented and other essential components of teaching and learning ( Shukla, 2019). Teaching and learning have always been based on what students, teachers, and policymakers believe. Their perspectives, experiences, logical arguments, and quasi-experiments enlighten the teaching and learning process. However, when teaching and learning are applied based on the faculties of the brain, they facilitate more effective and comprehensive student learning ( Shukla, 2019).

Several researchers examined the brain and its functions as ‘the primary learning organ.’ As a result, BBL emerged as one of the most effective learning methods, providing learners with excellent opportunities. According to the BBL theory, the brain’s plasticity (the ability to remodel itself for improved functionality) is maintained throughout life. El-Wakeel (2008) has confirmed that the structure and function of the brain are the foundations of this learning theory. In this sense, it can be stated that ‘learning will occur if the brain is not prevented from carrying out its usual functions.’

A massive shift has been achieved in neuroscience during the last three decades; the discovery of neuroplasticity, ‘the brain’s ability to selectively modify itself in response to specific repeated actions and experiences,’ proved that the brain retains its flexibility throughout life ( Cramer *et al*., 2011). Thus, neuroscientists have gathered reliable data through designing clinical studies that use double-blind, extensive, diverse, multi-age, multicultural groups of people to determine how humans learn ( Spears & Wilson, 2021). The way the brain functions has been identified to have a substantial impact on the most successful learning activities ( Wilson, 2013).

The last two decades witnessed several successful examples of practicing BBL in all levels of education in numerous subject areas. Academics from prominent universities worldwide have incorporated this knowledge in their classrooms and research, based explicitly on conclusions from neuroscience research ( Spears & Wilson, 2021).

### Brain-based learning for creative disciplines

Disciplines that are based on creativity and visual creation, such as design and visual arts, maybe served most by BBL methods and strategies. Caine and Caine (1995) stated that BBL stresses the importance of ‘patterning,’ which is that the brain easily learns logic and creates meaning because it relates, integrates and connect information and builds upon it, as humans resist learning unrelated information, this may be seen in design and visual arts education. Another point that relates BBL to design and visual arts education is that BBL stresses the principle that the brain is a ‘parallel processor,’ which performs complex cognitive and creative functions simultaneously and in a nonlinear way.

Learning design and art requires exceptional capabilities and skills; students must manipulate knowledge, information, and experiences they gain, relate, connect and convert them into creative, aesthetical, and functional ideas and meaningful outcomes to solve a design problem. This manipulation is the core of creativity, and it takes varying lengths of time. It also requires concentration and effort, leaving the student emotionally involved and stressed.

BBL can help design and visual art students understand how their brains work, think, and perceive; it can also facilitate the development of creative skills with reduced frustration ( El-Wakeel, 2020). Therefore, BBL provides a biologically based framework for teaching and learning in the context of design and visual arts that explains to students the concepts behind learning; essentially learning how to learn. This meta-concept encompasses a diverse range of strategies that reduce the amount of stress in the classrooms and achieve educational goals easier and faster. Yet, educationalists, especially in the field of design and visual arts, have not fully utilized results from neuroscience studies into BBL methodologies.

Edwards (2002) discussed the increasing number of research and activities related to creativity throughout the next century, where the concept of creativity evolves from simply a process of solving problems (that continually arise in human life) into a meta-process of generating new ideas about the process of searching and identifying the issues to solve where no one else has perceived.

Despite the relevance of BBL to design and arts education as creativity-inspired disciplines, yet there is scarcity in published research that has made the proper connection between them.

A very few studies were found that incorporated BBL principles into learning in the field of ‘Arts’ in general but not particularly in design and visual arts. For example, Betty Edwards from California State University and Howard Gardner from Harvard University are among the very few authors who incorporated BBL into the design and visual arts. Edwards’s book “ *The New, drawing on the Right Side of the Brain*” (2002) is a brilliant approach to recent developments in brain-based research that relate to developing drawing skills. In addition, Gardner’s book “ *Art Education and Human Development*” (1991) is another pioneering work that links art education with several brain capabilities.

A more recent pioneering study focusing on ‘Design’ applied BBL in studio-based design courses for three years, aiming to support, develop, and improve teaching strategies and learning processes ( El-Wakeel, 2008). The study applied 12 principles of BBL in designing courses and observing students’ performance to modify pedagogical strategies accordingly. The result isolated six BBL principles directed towards enhancing the creativity progress stages in ‘Arts and Design’ and enhancing the students’ creative capabilities in a much shorter time and less stressful way. In 2020, El-Wakeel argued that creativity is an exceptional brain progressive capability that needs a direct simultaneous response from the brain towards the body. The study added that BBL offers design and art educators a perfect chance to understand the human brain’s physiology and performance and then convert this knowledge into educational tools and principles. This was a new integrated view of the learning process for the learner. The study claimed that students could develop their skills, activate their creative brain capabilities, and control the stages of their creative process by applying this instructional approach to design freshmen students ( El-Wakeel, 2020).

The necessity for more specialized studies and application of BBL in design and visual arts motived the authors to perform a bibliometric analysis to identify the gaps, trends, subject development, and growth in the published body of literature on BBL in design, visual arts, and creativity-based subjects. Identifying such gaps will help direct more focused and applicable future research.

### Research aim and objectives

Aiming to identify the gaps in the current literature that relates brain-based learning to creative subjects such as design and visual arts. This research therefore carries out the following objectives:

1. Map and visualize Scopus indexed BBL literature published in the past twenty years, using bibliometric methods and scientometric analysis tools to evaluate yearly research growth and trends, citation structure, topics of the most productive publication sources, prolific authors and their disciplines, authorship patterns, contributing organizations and countries, and collaborating funding bodies.
2. To critically analyze patterns of progression and evolution of research in BBL according to their keywords, topics, themes and titles in the studied time span.
3. To detect the actual gaps and decline in BBL research in disciplines related to creativity, design and visual arts based on the aforementioned analysis.

## Methods

### Data selection and method

The bibliometric method (a quantitative evaluation for analyzing bibliographic data) was employed to assess the research performances of brain-based learning in terms of creativity-based subjects such as design and visual arts. Although this methodology of determining published research literature has been around for a long time, however, it became more prevalent with the introduction of large-scale bibliographic databases (e.g., Scopus, Web of Science, PubMed). The method used in this research presents the scientific landscape of annual growth trends, productive authors, actively participating countries, organizations, and collaborative contributors to the global scientific literature, with stress on investigating the evolution of themes and area topics studied in BBL research. Data was collected on 13 ^th^ June 2021 at Imam Abdulrahman bin Faisal University in Dammam, Saudi Arabia, from the Scopus database. The topics ‘design’ and ‘creativity’ were used in the initial search and then refined by the term ‘brain-based learning,’ this allowed the retrieval of records used in this study, the following search strategy was applied:

(TITLE-ABS-KEY (“Design”) OR TITLE-ABS-KEY (“Creativity”) AND (“brain-based learning”)

### Data retrieval and tools

The data was downloaded in BibTex, RIS format, and CSV format. Microsoft Excel, scientometric and bibliometric tools, such as Bibexcel ( Persson *et al.*, 2009), Biblioshiny ( Massimo & Corrado, 2019), and VOSviewer ( van Eck & Waltman, 2010), were used to analyze the data and visualize the results.

## Results

For the topics ‘design’ and ‘creativity,’ 5,980,330 records were retrieved; when refined by ‘brain-based learning,’ only 186 documents were retrieved. Those were the bases of this study; the results included 175 research papers that mentioned the word ‘design,’ however, the term ‘design’ is a vast word that yields several interpretations and relates to processes within many disciplines covering several aspects of the term, e.g., ‘syllabus design’ and ‘instructional design.’ Narrowing the search down, just 57 papers focused on ‘design education.’ Among those, only 3 research articles were directly related to ‘design and visual arts.’ These numbers reflect the huge gap in the BBL research that is directed towards topics in creativity, design and visual arts education.

According to the overall results, 454 authors transcribed the identified 186 research, spread in 128 sources during 2001-2021 (see Table 1). As shown, 8745 papers were cited to produce the 186 publications. The average number of publications was 5.39, while the average number of citations per document was 0.6875, and 5.446 was the average number of citations per document observed. The authors used 466 keywords in 186 publications. Single-author documents were 50, with averages of an author per document 2.44, co-authors per document 2.67, and a collaboration index of 3.01.

**Figure 1.**
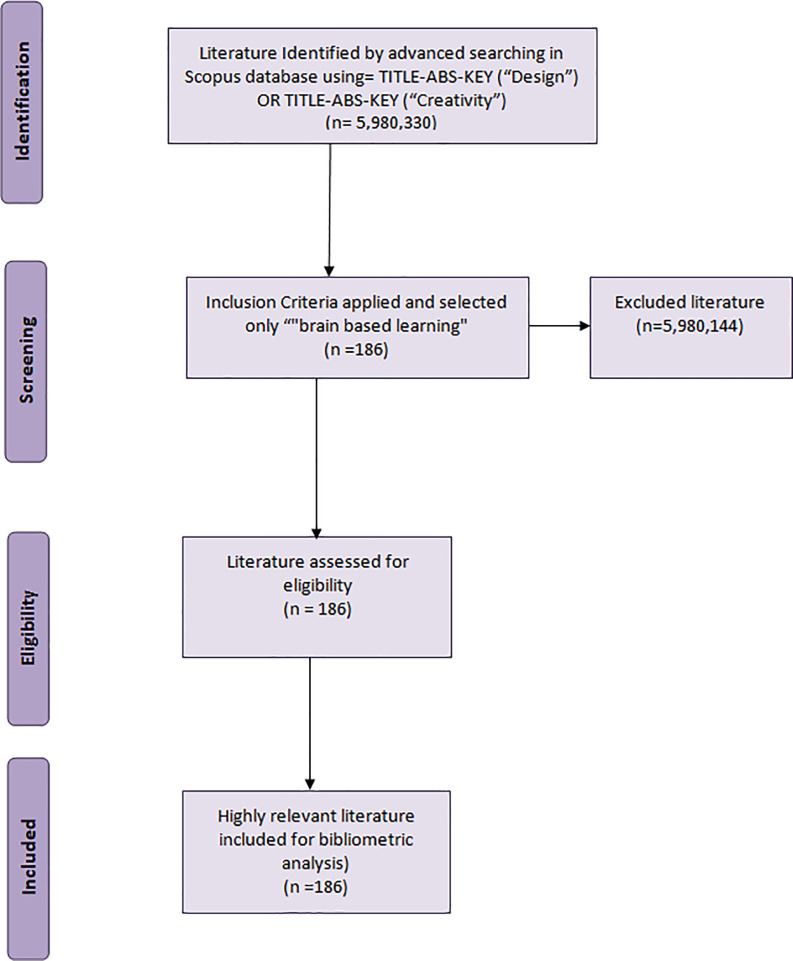
PRISMA flow diagram for data extraction.

**Table 1.** Overall results of brain-based learning research literature between 2001 and 2021.

Description	Results
**Main information about data**	
*Timespan*	2001:2021
*Sources (Journals, Books, etc.)*	128
*Documents*	186
*Average years from publication*	5.39
*Average citations per document*	5.446
*Average citations per year per doc*	0.6875
*References*	8745
**Document contents**	
*Keywords Plus (ID)*	673
*Author's Keywords (DE)*	466
**Authors**	
*Authors*	454
*Author Appearances*	496
*Authors of single-authored documents*	45
*Authors of multi-authored documents*	409
**Authors collaboration**	
*Single-authored documents*	50
*Documents per Author*	0.41
*Authors per Document*	2.44
*Co-Authors per Documents*	2.67
*Collaboration Index*	3.01

### Annual production trends and citation structure of brain-based learning research

The first research paper published in 2001 did not receive any citations; similarly, there were no publications in 2002. Remarkably, one paper was published in the year 2003 that received 66 citations. The average annual growth rate for the twenty years is about 9.3 publications. The literature production from 2001 to 2016 was below 15 publications; later an impressive research growth rate was observed in the recent five years (2017-2021). The year 2019 recorded the highest in research outcomes (29 publications, 35 citations), followed by 2020 (26 publications, 16 citations) 2018 (21 publications, 44 citations). The year 2021 recorded 11 publications with zero citations until 13 ^th^ June 2021. Table 2 displays the impact of citations over the twenty years (2001-2021), a total of 1013 citations were recorded for the 186 publications. The average yearly citation growth is stated as 50.65 citations. The highest number of citations were recorded in 2015 (128 citations) for only 14 publications, followed by 2016, 100 citations for nine publications. The analysis indicates a slow growth of publications in the first ten years, resulting in a higher citation rate per publication. The interest in the subject began to grow rapidly in the next ten years, with some fluctuations in the annual growth of publications and the impact of citations on BBL research.

**Table 2.** Annual publications (NP) and citations (TC) between 2001 and 2021 for brain-based learning research.

Year	NP	TC	Citation sum within h-core	h-index
2001	1	0	0	0
2003	1	66	66	1
2004	4	36	36	4
2005	1	5	5	1
2006	5	31	30	3
2007	1	3	3	1
2008	3	57	57	3
2009	7	84	82	3
2010	2	84	84	2
2011	10	99	93	4
2012	13	95	85	3
2013	5	40	38	3
2014	7	55	49	4
2015	14	128	115	6
2016	9	100	88	3
2017	16	35	27	3
2018	21	44	30	4
2019	29	35	20	4
2020	26	16	7	2
2021	11	0	0	0

* NP=Number of papers.

** TC=Total citation.

### Forms of publications in brain-based learning

Table 3 shows that the forms of publications most favored by authors in BBL research were journaled articles (96 papers, 649 citations), followed by conference paper (56 publications, 38 citations), then books (11 publications, 115 citations), book chapter (11 publications, 13 citations), and then review (with 7 publications and 115 citations). The short survey, note, and letter were the least preferred form for BBL research, with one paper each. The authors preferred form of publications agrees with the results found by Ansari *et al*. (2021) and Rahaman *et al*. (2021), where journal papers have a higher reach rate and ensure dissemination of results.

**Table 3.** Form of research publications on brain-based learning.

Rank	Form of research	NP	TC	Citation sum within h-core	h-index
1	*Journal Article*	96	649	443	14
2	*Conference Paper*	56	38	17	4
3	*Book*	11	115	102	4
4	*Book Chapter*	11	13	7	2
5	*Review*	7	115	114	5
6	*Conference Review*	2	0	0	0
7	*Note*	1	70	70	1
8	*Short Survey*	1	10	10	1
9	*Letter*	1	3	3	1

### Productive sources

The top ten sources of BBL research are listed in Table 4; looking closely at the results, it was pointed out that only three sources have more than five publications. *Journal of physics: conference series* (Q4) was acknowledged as the most relevant source for published BBL research with 22 publications and 17 citations, followed by the *European Journal of Social Sciences* (Q4) and then *Lecture Notes in Computer Science* (Q3) with three publications each and 11, and 2 citations, respectively. *International Journal of Advanced Computer Science and Applications* (Q3), *Journal on Mathematics Education* (Q2), *ACM International Conference Proceeding Series*, and *Procedia - Social and Behavioural Sciences* with three publications each having 5, 26, 3, and 15 citations, respectively. *Energy Education Science and Technology Part B: Social and Educational Studies*, *Journal of Cardiothoracic and Vascular Anesthesia* and *Proceedings - IEEE Symposium on Computers and Communications* came tenth in the top ten list with two publications each. Half of the leading sources belonged to the UK, followed by the USA (2), Germany, Indonesia, and Turkey each had only 1 source. It is observed from this result that none of the sources identified in the top ten for BBL research relate to design or visual arts specialties, so the authors had a closer look at all of the 186 publications, this revealed that most sources were within the fields of physics, computer science, education, behavioural sciences, etc., while there were only 3 sources that were directly related to design and visual arts studies and/or education, and they were not detected in the top ten results. This shows that there is a very slim opportunity for researchers to publish in platforms dedicated to this field which explains the extended literature gap in this area.

**Table 4.** Top ten most productive sources of brain-based learning research.

Rank	Source	NP	TC	Q	Country	Publisher	h_index	g_index	m_index	PY_start
1	Journal of Physics: Conference Series	22	17	Q4	UK	IOP pub	3	3	0.75	2018
2	European Journal of Social Sciences	5	11	Q4	UK	Euro Journals	2	3	0.18	2011
3	Lecture Notes in Computer Science	5	2	Q3	Germany	Springer	1	1	0.10	2012
4	International Journal of Advanced Computer Science And Applications	3	5	Q3	UK	Sci & Info org	1	2	0.33	2019
5	Journal on Mathematics Education	3	26	Q2	Indonesia	Sriwijaya Univ	3	3	0.75	2018
6	ACM International Conference Proceeding Series	3	3	NA	USA	ACM	1	1	0.11	2013
7	Procedia - Social and Behavioral Sciences	3	15	NA	UK	Elsevier BV	1	2	0.08	2009
8	Energy Education Science and Technology Part B: Social and Educational Studies	2	4	Q4	Turkey	Sila Science	2	2	0.20	2012
9	Journal of Cardiothoracic and Vascular Anesthesia	2	28	Q2	UK	W.B. Saunders Ltd	2	2	0.25	2014
10	Proceedings - IEEE Symposium on Computers and Communications	2	3	NA	USA	IEEE	1	1	0.10	2012

### Productive authors

The top ten most prolific authors of BBL research are listed in Table 5. Chaijaroen, S. (Khon Kaen University) is the highest publishing author with 22 publications and 7 citations. Howard-Jones, P.A. (University of Bristol), Yelamarthi, K. (Central Michigan University), and Abidin, SRZ. (University of Technology Mara), each has three publications and 32, 51, and 4 citations, respectively. Ashaari, N.S., Ausburn, L.J., Bose, R., Drake, E., Hendriana, H., and Hess, P.E. ranked tenth of the list, with two publications each and 4, 5, 28, 50, 19, and 28 citations, respectively. Yelamarthi, K. was identified as the most impactful author with 51 citations for three publications, followed by Drake, E. with 50 citations for two publications, and Howard-Jones, P.A. with 32 citations for three publications. The table also reveals that 50% of the top ten published authors are from the USA, followed by Malaysia (two authors), Thailand, Indonesia, and the UK are home to one author each.

**Table 5.** Top ten prolific authors in brain-based learning research between 2001 and 2021.

Rank	Author	Affiliations	Country	NP	TC	h_index	g_index	m_index	PY_start
1	Chaijaroen, S.	Khon Kaen University	Thailand	6	7	2	2	0.18	2011
2	Howard-Jones, P.A.	University of Bristol	UK	3	32	2	3	0.14	2008
3	Yelamarthi, K.	Central Michigan University	USA	3	51	2	3	0.29	2015
4	Abidin, S.R.Z.	University Technology Mara	Malaysia	3	4	1	2	0.33	2019
5	Ashaari, N.S.	University Kebangsaan Malaysia	Malaysia	2	4	1	2	0.33	2019
6	Ausburn, L.J.	Oklahoma State University	USA	2	5	2	2	0.33	2016
7	Bose, R.	Harvard Medical School	USA	2	28	2	2	0.25	2014
8	Drake, E.	Central Michigan University	USA	2	50	2	2	0.29	2015
9	Hendriana, H.	Institut Keguruan dan Ilmu Pendidikan	Indonesia	2	19	2	2	0.50	2018
10	Hess, P.E.	Harvard Medical School	USA	2	28	2	2	0.25	2014

When looking at the specialties of authors with high citations, they happened to be specialized in the fields of engineering and technology education, neuroeducation, neuropsychological concepts, computer science, and medicine; these publications appear to have more acceptable and useable results. On the other hand, the low impact of authors specialized in design and visual arts education raises the question on why researchers, academics, and educationalists in design disciplines are not directed towards liking BBL to design education despite the significance of this topic and its high relevance to enhancing design students’ knowledge acquisition and creativity.

### Pattern of authorship

**Figure 2.**
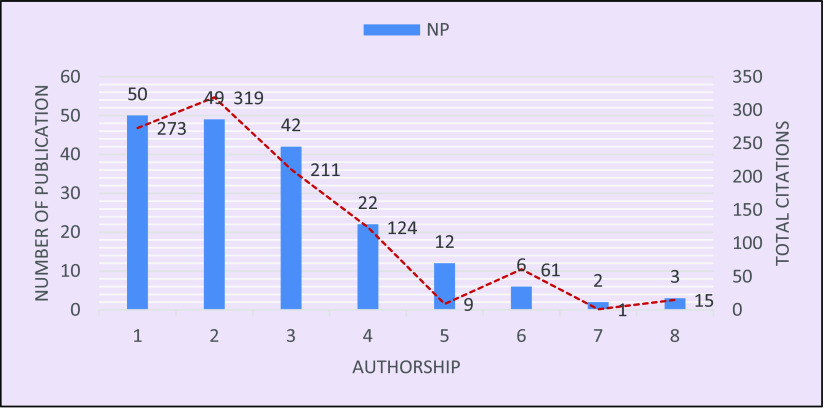
Authorship pattern in brain-based learning.

The authorship pattern is visualized in Figure 2, which shows that out of 186 publications, only 50 papers represent single authorship, and these 50 received 273 citations. Double authorship produced 49 papers and received 319 citations, followed by triple authorship, which contributed to 42 papers, with 211 citations, and four-authored research yielded 22 papers with 124 citations. A much smaller number of publications, 23, included five or more authors with 86 citations in total. The visualization infers that the more authors contribute to a research, the more general it may be, hence it is cited less. Another inference is that there is a lower chance of collaboration due to the scarcity of researchers specialized in this topic. A similar result on the authorship pattern was reported by Rahaman *et al*. (2021).

### Productive organization

The top ten most beneficial organizations of BBL research are displayed in Table 6. Khon Kaen University in Thailand produced 13 papers, followed by Universitas Pendidikan Indonesia with eight papers. Central Michigan University in the United States produced 7 papers, Universitas Sultan Ageng Tirtayasa in Indonesia, and Universiti Kebangsaan Malaysia with 5 papers each. The Harvard Medical School, Universitas Negeri Yogyakarta, University of Bristol, and the University of Oklahoma contributed to 4 publications each. Imam Abdulrahman Bin Faisal University ranked last in the top ten, contributing to 2 papers of BBL research. When investigating the titles of these organizations’ publications, it was found that research from the highest-ranking organizations focused on mathematics, physics, and computer education, while the less-ranking organizations produced research on neuroscience, engineering, mathematics, and design education. Therefore, there is more room for contribution in the topics related to creative disciplines.

**Table 6.** Top ten productive organization in brain-based learning research.

Rank	Affiliations	Country	NP
1	Khon Kaen University	Thailand	13
2	Universitas Pendidikan Indonesia	Indonesia	8
3	Central Michigan University	USA	7
4	Universitas Sultan Ageng Tirtayasa	Indonesia	5
5	Universiti Kebangsaan Malaysia	Malaysia	5
6	Harvard Medical School	USA	4
7	Universitas Negeri Yogyakarta	Nigeria	4
8	University of Bristol	UK	4
9	University of Oklahoma	USA	4
10	Imam Abdulrahman Bin Faisal University	Saudi Arabia	2

### Productive country

Indonesia was acknowledged as the most productive country in BBL research in Table 7, producing 48 papers and receiving 61 citations, followed by the United States (39 papers and 405 citations), Turkey (16 publications and 44 citations), Thailand (14 publications and 17 citations), and Malaysia (10 and 17 citations). China and Saudi Arabia ranked tenth on the list with 4 publications each, having 5 and 3 citations, respectively. In terms of order of citations, the United States scored the highest number of citations (405), followed by the United Kingdom (229 citations) and Indonesia (61 citations).

**Table 7.** Top ten productive countries in brain-based learning research.

Rank	Country	NP	TC
1	Indonesia	48	61
2	United States	39	405
3	Turkey	16	44
4	Thailand	14	17
5	Malaysia	10	17
6	United Kingdom	10	229
7	South Africa	5	4
8	Taiwan	5	56
9	China	4	5
10	Saudi Arabia	4	3

### Analysis of author keywords

**Figure 3.**
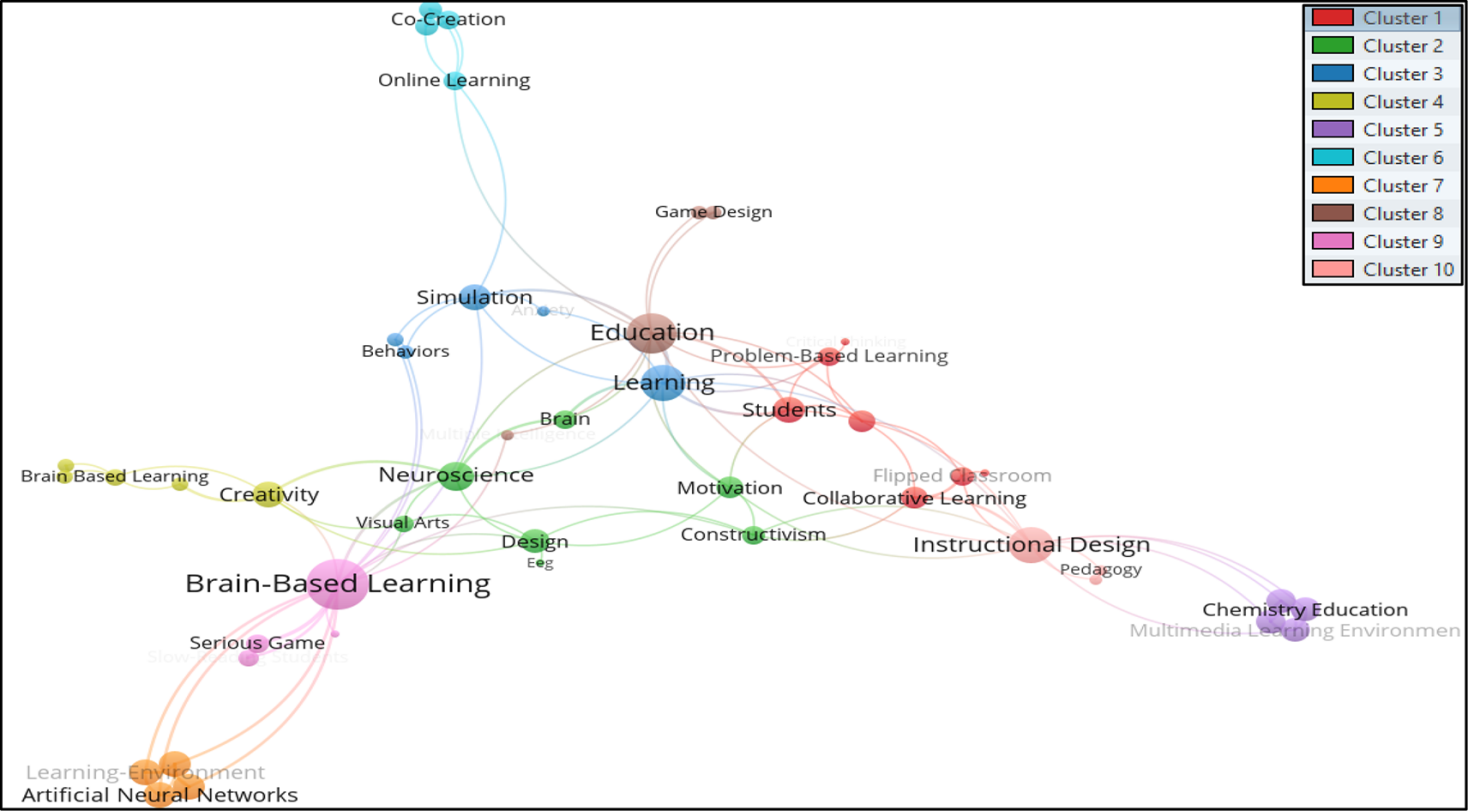
Visualization of authors’ keywords using VOSviewer Software.

The keywords set by authors in their published work are considered to be more precise and better express the research’s scope than those set by the publisher. The co-occurrences of authors’ keywords are assessed in this paper to identify BBL research trends; therefore, a minimum of two co-occurrences are considered; this is similar to the type of analysis done by Rahaman *et al*. (2021). Thus, from the total of 466 keywords, only 49 met the thresholds. The total strength of the co-occurrence links with other keywords was calculated for each of the 49 keywords where the total link strength were selected. Hence 47 keywords, 10 clusters, 102 links, and 139 total link strengths were observed. The 10 clusters are differentiated through a colour code shown in Figure 3, where authors’ keywords are visualized using VOSviewer software.

The authors’ keywords that appeared the most in the studied BBL research were; Brain-Based Learning, Education, Instructional Design, Learning, Creativity, Brain, Neuroscience, Achievement, Brain-Based Learning, and Collaborative Learning. Keywords are grouped in the following clusters:

*Cluster 1 consists of seven author keywords*: Active Learning, Collaborative Learning, Critical Thinking, Flipped Classroom, Mathematics Education, Problem-Based Learning, and Students. The most common (n = 4) author keywords in this cluster are Collaborative Learning and Flipped Classrooms.

*Cluster 2 consists of seven author keywords*: Brain, Constructivism, Design, EEG, Motivation, Neuroscience, and Visual Arts. The keywords Brain and Neuroscience were found to be the most occurring (five times each) author keywords in this group.

*Cluster 3 includes the five author keywords*: Anxiety, Behaviors, Learning, Scaffolding, and Simulation. Learning (n=7) was found as a highly occurring author keyword in this group.

*Cluster 4 includes five author keywords*: Achievement, Brain-Based Learning, Creative Thinking, Creativity, and Retention. Creativity (n=6) was noted as the highest occurring author keyword in this cluster.

*Cluster 5 comprises four author keywords*: Chemistry Education, Constructivist Learning Environment, Multimedia Learning Environment, and Scientific Thinking. All of the author’s keywords appeared at least twice in this cluster.

*Cluster 6 consists of four author keywords*: Co-Creation, Online Learning, Student Engagement, and Virtual Reality. Online learning appeared the most in this cluster (n=3).

*Cluster 7 includes four author keywords*: Artificial Neural Networks, Face-To-Face Tutoring, Learning-Environment, and Mental Stimulation. All the chosen keywords appear at least twice.

*Cluster 8 includes the following keywords*: education, game design, multiple intelligence, and serious games. Education (n=10) pointed out as the most occurred author keyword in cluster 8.

*Cluster 9 consists of four author keywords*: Brain-Based Learning, Elementary School, Serious Game, and Slow-Reading. The phrase Brain-based Learning (n = 14) highly appeared keyword in the cluster.

*Cluster 10* includes the keywords: Instructional Design, Pedagogy, and Problem-Solving. Instructional design (n=8) was found as the highest-appearing keyword in the cluster.

The previous mapping shows that the authors’ keywords used to search for BBL research may not include the subject area related to brain-based learning, but rather they mostly include pedagogical aspects teaching strategies, instructional design. Searching for keywords such as Design and Visual arts may not yield many results; however, the keyword Neuroscience was found in the same cluster as Design and Visual Arts. The occurrences were very low, but there is a direct association between these fields researchers must relate to. Creativity and Creative thinking are keywords that find somewhat relevant literature related to design disciplines.

### Mapping co-occurrence of all keywords

**Figure 4.**
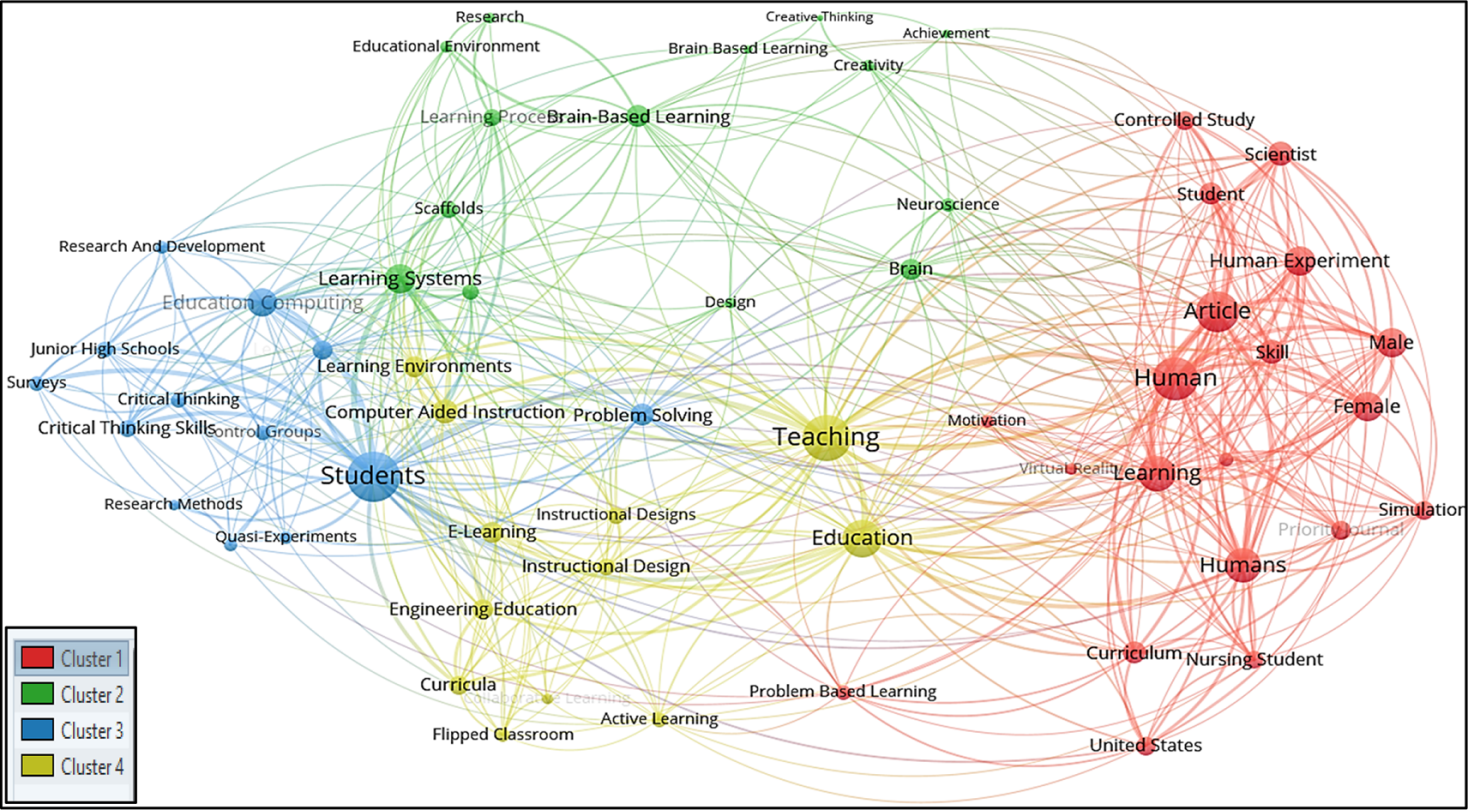
Visualization of all-keywords using VOSviewer Software.

For all keywords’ analysis, a minimum of four occurrences of all keywords were considered in this research; therefore, out of 1018 keywords, only 59 meet the thresholds. The total strength of the co-occurrence links with the other keywords was calculated for each of the 59 keywords, and the greatest total link strength was also calculated. Hence 59 keywords, 4 clusters, 527 links, and 1082 total link strength were observed. All 4 clusters were then differentiated into four different colours, as seen in Figure 4.

*Cluster 1 comprises 20 keywords*: article, controlled study, curriculum, female, human, human experiment, humans, learning, male, motivation, nursing student, online learning, priority journal, problem-based learning, scientist, simulation, skill, student, united states and virtual reality. Among the 20 keywords, the most occurred keywords were human and article with 15 and 14 frequency, respectively.

*Cluster 2 consists of 14 keywords*: achievement, brain, brain-based learning, brain-based learning, creative thinking, creativity, design, educational environment, learning achievement, learning process, learning systems, neuroscience, research, and scaffolds. Among the 14 keywords, the highest occurred keywords were brain-based learning and learning systems occurring 14 times each.

*Cluster 3 included 13 keywords*: control groups, critical thinking, critical thinking skills, education computing, junior high schools, learning models, physics, problem-solving, quasi-experiments, research and development, research methods, students, and surveys. The most occurred keywords in this group were students and education computing with 42 and 14 respectively.

*Cluster 4 represented 12 keywords*: active learning, collaborative learning, computer-aided instruction, curricula, e-learning, education, engineering education, flipped classroom, instructional design, learning environments, and teaching. Education and teaching were the most occurring keywords in this cluster, with 24 and 23 occurrences, respectively.

It is observed in Figure 4 that ‘creativity’ and ‘design’ in Cluster 2 occurred scarcely in the studied publications; the term ‘art’ and ‘visual art’ did not appear to occur in the all-keywords categories.

A comparison between the highly accruing ‘all-keywords’ and ‘authors’ keywords’ are listed in Table 8. It is noticed that the authors’ keywords are more precise than all keywords. It is also found that the appearance of the keywords such as ‘Brain-Based Learning’ is the same in all keywords and author keywords; however, it is on the top of the top-ten list of ‘authors’ keywords’ and the sixth in the list of ‘all keywords’ top-ten list. The keyword ‘Creativity’ appears fifth in the authors’ keywords list, whereas it does not appear in the all-keywords list’s top ten occurrences.

**Table 8.** Comparison between ‘authors keywords’ and ‘all-keywords’.

Rank	Authors keywords		All-keywords	
	Keywords	Occurrences	Keywords	Occurrences
1	Brain-Based Learning	14	Students	42
2	Education	10	Education	24
3	Instructional Design	8	Teaching	23
4	Learning	7	Human	15
5	Creativity	6	Article	14
6	Brain	5	Brain-Based Learning	14
7	Neuroscience	5	Education Computing	14
8	Achievement	4	Learning	14
9	Brain Based Learning	4	Learning Systems	14
10	Collaborative Learning	4	Humans	10

### Thematic evaluation by topic

**Figure 5.**
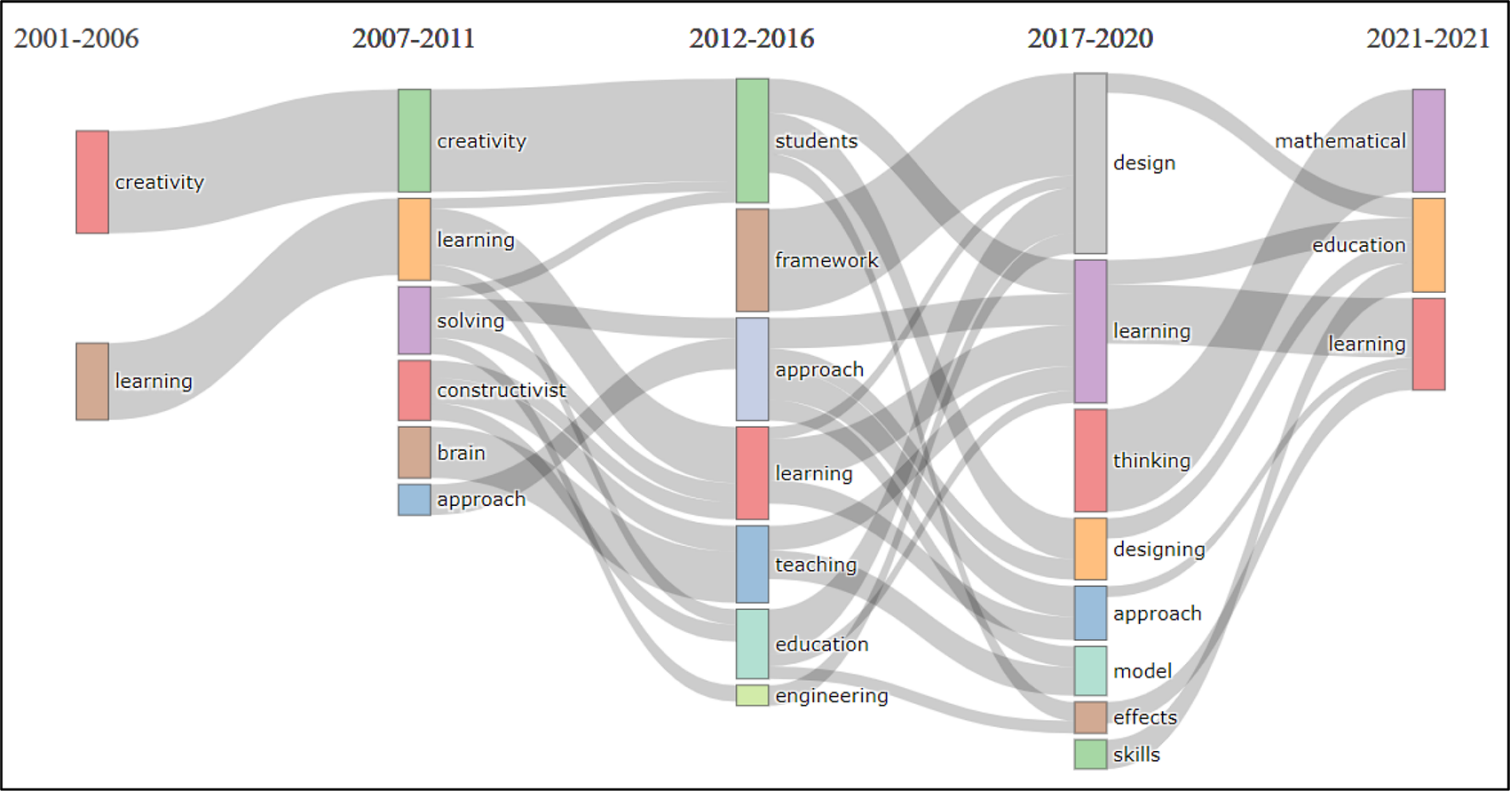
Visualization of thematic evolution analysis by topic using VOSviewer Software.

The thematic evolution analysis by topic was considered to investigate the stability, progression and/or regression in BBL research topics. For this type of analysis, a topic-model unigram was developed from 250 words with a minimum cluster frequency (5), and a time slice (4) with cutting years for each slice: 2006, 2011, 2016, and 2020 respectively, see Figure 5.

The unigram shows the most frequent topics, where it can be seen that the period 2001-2006 includes BBL research related merely to two topics which are ‘creativity’ and ‘learning’, these topics remain in the focus of BBL research until 2007. The development in the subject area can be noticed starting from 2007 up to 2012, where combinations of topics are noticed relating the initial titles ‘creativity’ and ‘learning’ to ‘students’ and ‘education’. While there are a number of emerging topics such as ‘solving-problems’, ‘constructivist’, ‘brain’ and ‘approach’ with consistent development. By 2012, research relating those topics to ‘approach’, ‘learning’, ‘teaching’, ‘education’ and ‘engineering’ were published.

The topic ‘framework’ emerged during the period 2012-2016, which mostly contributed to the development of research related to the topic ‘design’ in 2017-2020. However, this does not relate directly to ‘design and creativity’ or ‘design and art’, but rather to educational frameworks, engineering and curricula design, as shown in the Figure 5.

More interestingly, this unigram shows that several topics such as ‘thinking’, ‘designing’, ‘model’, ‘effect’ and ‘skills’ are not showing any relationship with the topic ‘design’, contrary to what may be assumed. The visualization also reveals that in 2017-2020, a few new topics have emerged such as ‘thinking’, and ‘skills’ which are strongly related to ‘mathematics’ and ‘education’. It is also worth mentioning that the topic ‘visual arts’ is missing from the unigram, meaning its frequency was not detected. The thematic evolution analysis substantiates that there is decline in the development of BBL research relating to the topics of design, visual arts, creativity and design education. Table 9 presents the number of occurrences

**Table 9.** Thematic analysis by topics and their number of occurrences.

From	To	Topics	Occurrences
Creativity–2001-2006	Creativity–2007-2011	**Creativity**	2
Learning–2001-2006	Learning–2007-2011	**Learning**	6
Approach–2007-2011	Approach–2012-2016	**Approach**	5
Brain–2007-2011	Teaching–2012-2016	**Brain; Classroom**	5
Constructivist–2007-2011	Education–2012-2016	**Science**	2
Constructivist–2007-2011	Learning–2012-2016	**Development**	2
Constructivist–2007-2011	Teaching–2012-2016	**Teaching**	3
Creativity–2007-2011	Students–2012-2016	**Creativity**	2
Learning–2007-2011	Education–2012-2016	**Education**	3
Learning–2007-2011	Learning–2012-2016	**Learning**	11
Learning–2007-2011	Students–2012-2016	**Effect**	2
Solving–2007-2011	Approach–2012-2016	**Creative**	2
Solving–2007-2011	Engineering–2012-2016	**Instructional; Achievement**	3
Solving–2007-2011	Learning–2012-2016	**Thinking; Model**	3
Solving–2007-2011	Students–2012-2016	**Students**	3
Solving–2007-2011	Teaching–2012-2016	**Based**	2
Approach–2012-2016	Approach–2017-2020	**Approach**	3
Approach–2012-2016	Designing–2017-2020	**Designing**	2
Approach–2012-2016	Learning–2017-2020	**School**	3
Approach–2012-2016	Model–2017-2020	**Creative**	2
Education–2012-2016	Design–2017-2020	**Education; Brain_Based**	8
Education–2012-2016	Effects–2017-2020	**Study**	3
Education–2012-2016	Learning–2017-2020	**Science**	3
Engineering–2012-2016	Approach–2017-2020	**Achievement**	2
Engineering–2012-2016	Design–2017-2020	**Engineering; Student**	4
Engineering–2012-2016	Model–2017-2020	**Flipped**	2
Framework–2012-2016	Design–2017-2020	**Framework**	2
Learning–2012-2016	Approach–2017-2020	**Environment; Scientific; Chemistry**	5
Learning–2012-2016	Design–2017-2020	**Design**	8
Learning–2012-2016	Learning–2017-2020	**Learning; Development**	20
Learning–2012-2016	Model–2017-2020	**Model**	3
Learning–2012-2016	Skills–2017-2020	**Multimedia**	2
Learning–2012-2016	Thinking–2017-2020	**Thinking**	4
Students–2012-2016	Approach–2017-2020	**Grade**	2
Students–2012-2016	Designing–2017-2020	**Educational; Elementary**	3
Students–2012-2016	Effects–2017-2020	**Creativity; Neuroscience**	3
Students–2012-2016	Learning–2017-2020	**Students; Effect**	5
Teaching–2012-2016	Learning–2017-2020	**Brain; Based**	4
Teaching–2012-2016	Model–2017-2020	**Teaching; Classroom**	5
Approach–2017-2020	Learning–2021-2021	**Approach**	10
Design–2017-2020	Education–2021-2021	**Education**	9
Designing–2017-2020	Education–2021-2021	**Elementary**	2
Effects–2017-2020	Education–2021-2021	**Teachers**	3
Effects–2017-2020	Learning–2021-2021	**Neuroscience; Study**	3
Learning–2017-2020	Education–2021-2021	**Students; Effect**	29
Learning–2017-2020	Learning–2021-2021	**Learning; Based; Development**	44
Skills–2017-2020	Education–2021-2021	**Skills; Mathematics**	10
Thinking–2017-2020	Education–2021-2021	**Thinking**	13
Thinking–2017-2020	Mathematical–2021-2021	**Mathematical; Improve**	10

### Most cited publications

The top ten highly cited publications of BBL research are sorted in Table 10. The 2010 article entitled “ *Using a games console in the primary classroom: Effects of ‘Brain Training’ programme on computation and self-esteem*” by Miller, D.J. and Robertson, D. P. was the most cited publication with 81 citations, followed by “ *The practical and principled problems with educational neuroscience*” by Bowers, J.S. with 70 citations (2016), “ *Participatory Action Research: creating an effective prevention curriculum for adolescents in the Southwestern US*” by Gosin *et al*. (2003) with 66 citations came afterward. “ *Linking Architecture and Education: Sustainable Design for Learning Environments*” (2009) by Taylor, A. (2009) with 60 citations and “ *A review of empirical evidence on scaffolding for science education*” by Lin *et al*., (2012) with 44 citations followed. The paper entitled “ *Variables Affecting Learning in a Simulation Experience: A Mixed Methods Study*” by Beischel, K.P. (2013) ranked tenth in the list with 29 citations. Table 9 also shows that most of the top ten cited papers were published between 2003 and 2016. The highest total citations per year (T.C./Year =11.67), as well as the highest normalized total citations (NTC=6.30), were reported in the article entitled “ *The practical and principled problems with educational neuroscience*” ( Bowers, 2016). From the titles of the publications, it is revealed that the most cited publications are directed towards using pedagogical strategies of BBL as well as applying different technological methods to enhance students’ knowledge acquisition and skills attainment in several disciplines. While one publication by Taylor (2009) focused on designing the learning environment to develop learning senses and perceptions of students, where the built environment becomes a teaching tool.

**Table 10.** Top ten most cited publications in brain-based learning between 2001 and 2021.

Rank	Title	Author	Year	Source	TC	TC/Year	NTC
1	“Using a games console in the primary classroom: Effects of 'Brain Training' programme on computation and self-esteem” ( Miller & Robertson, 2010)	Miller, D.J.	2010	*Br J Educ Technol*	81	6.75	1.93
2	“The practical and principled problems with educational neuroscience” ( Bowers, 2016)	Bowers, J.S.	2016	*Psychol Rev*	70	11.67	6.30
3	“Participatory Action Research: creating an effective prevention curriculum for adolescents in the Southwestern US” ( Gosin *et al*., 2003)	Gosin, M.N.	2003	*Health Educ Res*	66	3.47	1.00
4	“Linking Architecture and Education: Sustainable Design for Learning Environments” ( Taylor, 2009)	Taylor, A.	2009	*Book -UNM Press*	60	4.62	5.00
5	“A review of empirical evidence on scaffolding for science education” ( Lin *et al*., 2012)	Lin, T.C.	2012	*Int J Sci Math Educ*	44	4.40	6.02
6	“A Flipped First-Year Digital Circuits Course for Engineering and Technology Students” ( Yelamarthi & Drake, 2015)	Yelamarthi, K.	2015	*IEEE Trans Educ*	43	6.14	4.70
7	“A Modified Team-Based Learning Physiology Course” ( Persky & Pollack, 2011)	Persky, A.M.	2011	*Am J Pharm Educ*	42	3.82	4.24
8	“Neuroethics, Neuroeducation, and Classroom Teaching: Where the Brain Sciences Meet Pedagogy” ( Hardiman *et al*., 2012)	Hardiman, M.	2012	*Neuroethics*	38	3.80	5.20
9	“The effects of the computer-based instruction on the achievement and problem solving skills of the science and technology students” ( Serin, 2011)	Serin, O.	2011	*Turk Onl J Edu Tech*	35	3.18	3.54
10	“Variables Affecting Learning in a Simulation Experience: A Mixed Methods Study” ( Beischel, 2013)	Beischel, K.P.	2013	*West J Nurs Res*	29	3.22	3.63

### Country collaboration

**Figure 6.**
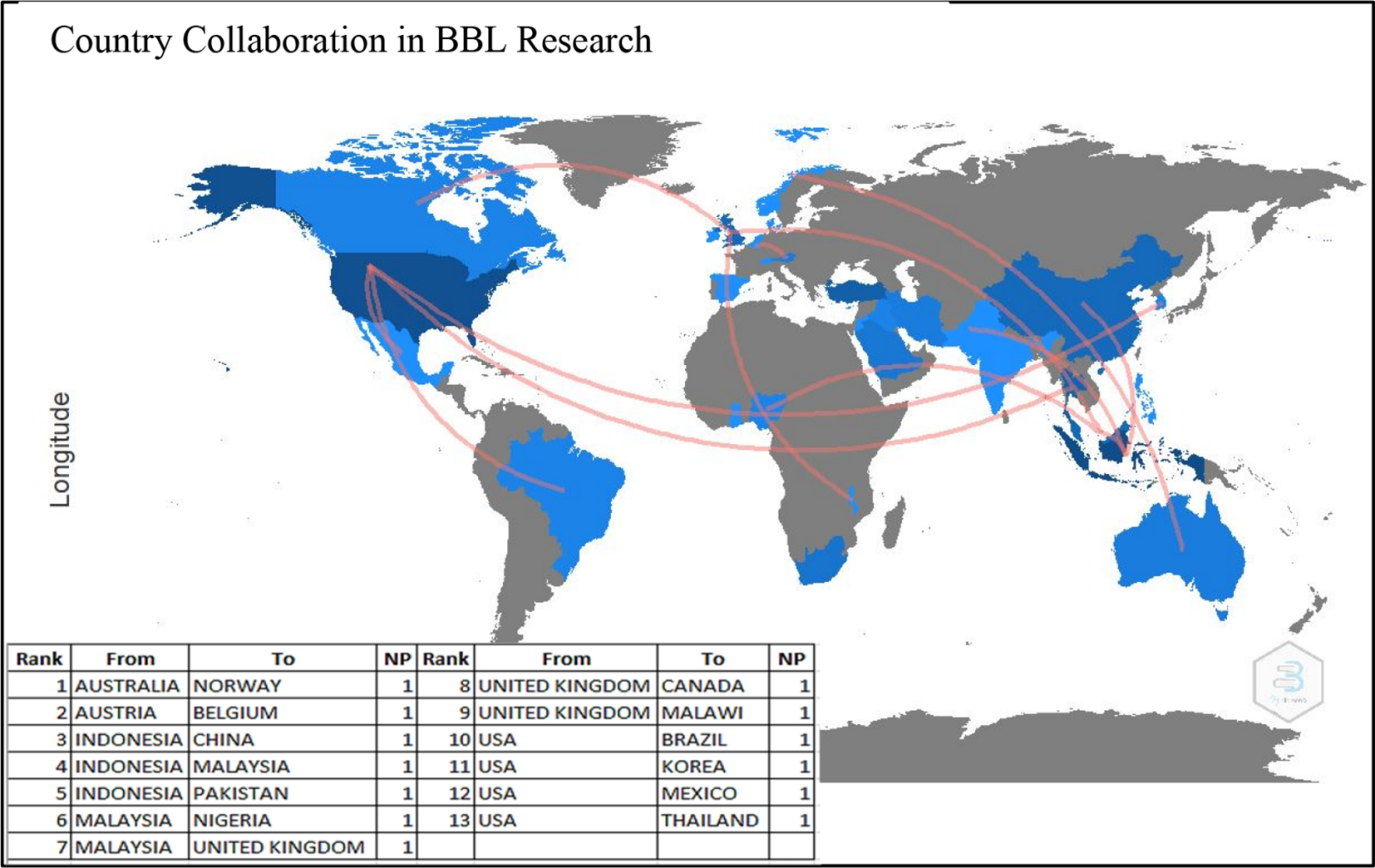
Country collaboration in brain-based learning research.

Figure 6 demonstrates the country collaboration patterns in producing research on BBL during 2001-2021. Surprisingly, all the listed 13 countries contributed to single partnerships each, i.e., Australia with Norway, Austria with Belgium, Indonesia with China, Indonesia with Malaysia, Indonesia with Pakistan, Malaysia with Nigeria, Malaysia with the UK, the UK with Canada, the USA with Brazil, the USA with Korea, the USA with Mexico and the USA with Thailand. The figure also shows that the United States collaborated the most with other countries (four collaborations). These results indicate that there is no particular direction or pattern of collaborative and joint research across countries.

### Most impactful funding agencies

**Figure 7.**
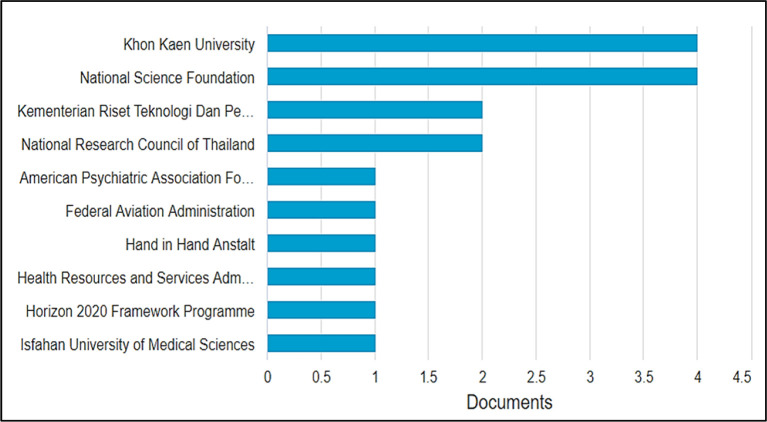
Top ten funding agencies with most research in brain-based learning.

As visualized in Figure 7, between 2001 and 2021, the top ten funding agencies that supported the highest number of researches in BBL were Khon Kaen University in Thailand and National Science in the United States with four funded publications each, followed by the Indonesian Riset Teknologi Dan Pendidikan Tinggi Republik Indonesia and the National Research Council of Thailand, each funding two publications. The American Psychological Association Foundation, the Federal Aviation Administration, the Hand in Hand Institute, the Health Resources and Services Administration, the Horizon 2020 framework program, and Isfahan University of Medical Sciences each agency financed only one publication. These results give insight into the active funding bodies interested in supporting BBL-related research, so researchers willing to develop outcomes and practical results can approach them. Another inference is made; the low number of funding agencies contributing to BBL research explains the low numbers of publications in this field which require developing instructional technology, apparatus, and setting up experiments and quasy-experiments, moreover, need high numbers of participants/learners to produce publishable and applicable outcomes.

## Conclusion

This research identified the relevance of brain-based learning in the field of design and arts education as it carefully considers how the brain learns, especially in creativity-based subjects.

However, the initial literature review indicated the absence of publications relating BBL pedagogical methodologies to design and visual arts education despite the associated relevance regarding these vital topics. The authors saw it as an opportunity to conduct an evidence-based bibliometric analysis for critical review and assessment of Scopus indexed published literature on brain-based learning during the past twenty years. The results demonstrated the growth trends in research, identified the productive authors, institutions, sources, authorship patterns, and visualized subject variation and keyword mapping, etc. The discussion of the analysed results revealed that there was a slow literature growth in the first ten years of the millennia; however, the topic received attention in the latter ten years. There was an interruption in the publication growth during 2013 and 2014 but was followed by rapid development until 2020. It was also evident that collaborative authorship was preferred over single authorship, while double authorship received more citations.

Comparison of authors’ keywords with all keywords indicated that the subject approach of author keywords is direct, more precise, and relatable to the topic. However, the keywords that appeared the most in the studied publications mainly focused on learning and education in general, instructional design and collaborative learning, neuroscience, and creativity. No keywords related to ‘design’ and ‘arts’ as a topic were found. This indicates that there is a large gap in the current body of literature that relates BBL to creativity-based disciplines such as design and visual arts despite the apparent relevance between them. In addition, the thematic evolution analysis carried out in this research substantiates that there is clear regression in the development of BBL research relating to the topics of design, visual arts, creativity and design education.

Therefore, to contribute to the development of BBL research in design and arts education, several stakeholders may play active roles in recognizing and incorporating BBL techniques in design education. Decision-makers and funding agencies should implement policies and direct their support to apply strategies that empower the role of BBL in academia and research. Academics, researchers, and educationalists in design and visual arts institutes are advised to direct their attention to BBL pedagogical activities and funded action research to benefit from neurosciences research on how the brain learns. This will increase students’ conscious learning, expand their imagination, and improve creativity and support multiple intelligences that develop their learning experience. This will also contribute to increasing the number of studies aiming at improving BBL methods to keep pace with the global challenges in education that we are currently facing.

## Data availability

### Underlying data

Zenodo: Brain-based learning, https://doi.org/10.5281/zenodo.6298928 ( El-Wakeel *et al*., 2022a).

This project contains the following underlying data:

• Data for brainbased Bibliometrix.xlsx

### Extended data

Zenodo: Brain-based learning in design and visual arts education, https://doi.org/10.5281/zenodo.6386705 ( El-Wakeel *et al*., 2022b).

This project contains the following extended data:

• PRISMA flow diagram.pdf
• PRISMA Checklist.pdf

Data are available under the terms of the Creative Commons Attribution 4.0 International license (CC-BY 4.0).
