# Supplementary material for: Brain-based learning in design and visual arts education: a bibliometric assessment of Scopus indexed literature
Source: F1000Res. 2023 Jul 3;11:402. Originally published 2022 Apr 8. [Version 3] doi: 10.12688/f1000research.110294.3 (PMC10390795; doi:10.12688/f1000research.110294.3)
Supplement: Peer review history

# Peer review: Reviewer response for version 3

**DOI:** 10.5256/f1000research.150148.r183618

**Published:** 2023-07-31

**Authors:** Nadeem Siddique

**Affiliations:** 1 Gad and Birgit Rausing Library, Lahore University of Management Sciences, Lahore, Pakistan

I am satisfied with the responses of the authors. No further suggestions from my side for revision.

Are the rationale for, and objectives of, the Systematic Review clearly stated?

Partly

Is the statistical analysis and its interpretation appropriate?

Yes

Are sufficient details of the methods and analysis provided to allow replication by others?

Partly

Are the conclusions drawn adequately supported by the results presented in the review?

Partly

Reviewer Expertise:

Bibliometrics, Scientometrics, systematic review, library automation

I confirm that I have read this submission and believe that I have an appropriate level of expertise to confirm that it is of an acceptable scientific standard.

# Peer review: Reviewer response for version 3

**DOI:** 10.5256/f1000research.150148.r183619

**Published:** 2023-07-04

**Authors:** Dr. Mohammad Rafiqur Rahman

**Affiliations:** 1 Department of Library and Information Science, Shri Jagdishprasad Jhabarmal Tibrewala University, Jhunjhunun, Rajisthan, India

The authors’ efforts have greatly improved the quality and clarity of the manuscript. The changes implemented have effectively addressed the reviewer's comments and strengthened the overall argument of the paper. The modifications made have enhanced the coherence of the arguments and provided a more comprehensive analysis of the topic. Thus making it worthy for indexing.

Are the rationale for, and objectives of, the Systematic Review clearly stated?

Yes

Is the statistical analysis and its interpretation appropriate?

Yes

Are sufficient details of the methods and analysis provided to allow replication by others?

Yes

Are the conclusions drawn adequately supported by the results presented in the review?

Partly

Reviewer Expertise:

Bibliometric, Scientometric, quantitative analysis.

I confirm that I have read this submission and believe that I have an appropriate level of expertise to confirm that it is of an acceptable scientific standard.

# Peer review: Reviewer response for version 2

**DOI:** 10.5256/f1000research.141376.r159804

**Published:** 2023-01-23

**Authors:** Dr. Mohammad Rafiqur Rahman

**Affiliations:** 1 Department of Library and Information Science, Shri Jagdishprasad Jhabarmal Tibrewala University, Jhunjhunun, Rajisthan, India

The topic chosen for the study is interesting, the purpose of the study is also clear, however,

 1) Authors should have looked for previous scientometric studies for BBL for insight of the topic. 

 2) All the indices have covered but the language-wise research production is missing. 

 3) Authors should have correlate their results with other bibliometric and scientometric studies to justify the findings. 

 4) In keyword analyses (author and all keywords) the selection of keywords for the analysis needs to write more clearly.

Are the rationale for, and objectives of, the Systematic Review clearly stated?

Yes

Is the statistical analysis and its interpretation appropriate?

Yes

Are sufficient details of the methods and analysis provided to allow replication by others?

Yes

Are the conclusions drawn adequately supported by the results presented in the review?

Partly

Reviewer Expertise:

Bibliometric , Scientometric, quantitative analysis.

I confirm that I have read this submission and believe that I have an appropriate level of expertise to confirm that it is of an acceptable scientific standard, however I have significant reservations, as outlined above.

# Response

**Published:** 2023-05-31

**Authors:** Hala Abdulmoneem Mahmoud El-Wakeel

**Affiliations:** Imam Abdulrahman Bin Faisal University, Saudi Arabia

1: Authors should have looked for previous scientometric studies for BBL for insight of the topic. 

 Reply: This study stands out as an original contribution, as no previous bibliometric study on this particular topic has been conducted to date. Extensive exploration of the existing literature has revealed no comparable research. Therefore, it is evident that this study is unquestionably novel and innovative.

 2: All the indices have covered but the language-wise research production is missing. 

 Reply: Thank you for suggesting language wise production, we have provided details of language wise research in methodology part.

 3: Authors should have correlate their results with other bibliometric and scientometric studies to justify the findings. 

 Reply:  Done as suggested in results section.

 4: In keyword analyses (author and all keywords) the selection of keywords for the analysis needs to write more clearly.

 Reply: Done as suggested.

# Peer review: Reviewer response for version 1

**DOI:** 10.5256/f1000research.121883.r153359

**Published:** 2022-11-02

**Authors:** Nadeem Siddique

**Affiliations:** 1 Gad and Birgit Rausing Library, Lahore University of Management Sciences, Lahore, Pakistan

This paper presents a bibliometric study conducted on 186 documents. The researchers did not explain the motivation behind this study. How would the study be helpful for the relevant audience, and what is the study’s significance? The methodology has some flaws. I could not retrieve the same number of records by using the query mentioned in the methodology section. I have retrieved only 51 documents by using the query in different ways in the Scopus database. The researcher did not provide details about the irrelevant and duplicate documents. The researchers should compare the previous research with their findings.

Are the rationale for, and objectives of, the Systematic Review clearly stated?

Partly

Is the statistical analysis and its interpretation appropriate?

Yes

Are sufficient details of the methods and analysis provided to allow replication by others?

Partly

Are the conclusions drawn adequately supported by the results presented in the review?

Partly

Reviewer Expertise:

Bibliometrics, Scientometrics, systematic review, library automation

I confirm that I have read this submission and believe that I have an appropriate level of expertise to confirm that it is of an acceptable scientific standard, however I have significant reservations, as outlined above.

# Response

**Published:** 2022-11-20

**Authors:** Hala Abdulmoneem Mahmoud El-Wakeel

**Affiliations:** Imam Abdulrahman Bin Faisal University, Saudi Arabia

The researchers did not explain the motivation behind this study.

**It is mentioned at the end of the introduction and conclusion.**

 How would the study be helpful for the relevant audience, and what is the study’s significance?

**It is mentioned in the introduction and conclusion.**

 The methodology has some flaws. I could not retrieve the same number of records by using the query mentioned in the methodology section. I have retrieved only 51 documents by using the query in different ways in the Scopus database.

**The error shows if the query copy and paste into the search as some unnecessary brackets appear, instead type the query in advance search which now showing 238 results as on 21/11/2022.**

 The researcher did not provide details about the irrelevant and duplicate documents.

**It is mentioned below the search query.**

 The researchers should compare the previous research with their findings.

**Unfortunately, there are no previous scientometric studies found on this topic, hence, it motivated researchers to conduct one.**

# Peer review: Reviewer response for version 1

**DOI:** 10.5256/f1000research.121883.r130165

**Published:** 2022-04-19

**Authors:** Aidi Ahmi

**Affiliations:** 1 Tunku Puteri Intan Safinaz School of Accountancy, Universiti Utara Malaysia, Sintok, Malaysia

Brain-based learning seems a good topic to be explored using bibliometric analysis. However, I have major concerns about the method of this study that will reflect the whole paper as below:

 The following search:

 TITLE-ABS-KEY (“Design”) OR TITLE-ABS-KEY (“Creativity”) AND (“brain-based learning”)

 will only reveal all documents that have keywords design and creativity in TITLE-ABS-KEY and “brain-based learning” anywhere in the text. So, the results only show all documents that met these criteria and NOT the articles related to the topic of “brain-based learning”.

 The screening process seems to have not been conducted. Although Figure 1 shows the screening process, it is actually a filtering process and NOT a screening process. The authors should look one by one at the title of the document to make sure all the documents being analysed are really about “brain-based learning”. Based on the dataset, it seems many documents are NOT about the topic of the study "Brain-based learning in design and visual arts education". In other words, the documents being analysed are totally NOT about "Brain-based learning in design and visual arts education".

 The authors have to make sure that all the documents being analysed are really about the topic or at least related to the topic of the study. The screening and cleaning process should be conducted for each of the documents before the data is downloaded from the Scopus database. Any irrelevant documents should be removed in order to make sure that all documents are really about the topic of the study and not about something else.

 Due to this, I can say that all the analyses conducted are considered invalid.

 The authors should re-design the search query to only gathered the documents related to "brain-based learning".

Are the rationale for, and objectives of, the Systematic Review clearly stated?

Partly

Is the statistical analysis and its interpretation appropriate?

Not applicable

Are sufficient details of the methods and analysis provided to allow replication by others?

No

Are the conclusions drawn adequately supported by the results presented in the review?

No

Reviewer Expertise:

Bibliometric analysis.

I confirm that I have read this submission and believe that I have an appropriate level of expertise to state that I do not consider it to be of an acceptable scientific standard, for reasons outlined above.

# Response

**Published:** 2022-05-10

**Authors:** Hala Abdulmoneem Mahmoud El-Wakeel

**Affiliations:** Imam Abdulrahman Bin Faisal University, Saudi Arabia

The authors appreciate the reviewer for reviewing the study and valuable suggestions.  The authors have no issues re-designing the search query to gather the data; however, they disagree that all the analyses conducted are considered invalid since the authors ensured a closer look at each document and ensured that all the documents are related to BBL.

# Response

**Published:** 2022-05-29

**Authors:** Hala Abdulmoneem Mahmoud El-Wakeel

**Affiliations:** Imam Abdulrahman Bin Faisal University, Saudi Arabia

The research observed that there is a decline in the development of BBL research which is related to the topics of design, visual arts, creativity, and design education, despite the fact that BBL methods are strongly beneficial to those fields and provide significant advantages to students learning awareness and performance especially for enhancing creative design skills (as identified in the literature review).

 The research aimed to identify the gaps in the current literature that relates brain-based learning to creative subjects such as design and visual arts, in order to identify the nature of such research and its topics, a refined search strategy is conducted and shown on page 4:

- The topics ‘design’ and ‘creativity’ were used in the initial search and then refined by the term ‘brain-based learning,’ this allowed the retrieval of records used in this study; the following search strategy was applied:
  (TITLE-ABS-KEY (“Design”) OR TITLE-ABS-KEY (“Creativity”) AND (“brain-based learning”)
  The research then conducts thematic analysis and identifies the topics of each research and finds that very few research apply BBL in design education specifically, and finally recommends educators and researchers to direct their
  attention to BBL pedagogical activities and funded action research to benefit from neurosciences research on how the brain learns. This will increase researcher in the field of design and visual arts education that utilizes BBL methods to enhance students' learning and to keep pace with the global challenges in education.

 

 The following parts of the research describe this:  

**The abstract** (p.1):

 Despite the significance to apply brain-based learning strategies in design and visual arts education to boost students’ knowledge and creative skills, the findings show a decline in quantities and growth patterns in brain-based learning research directed towards design disciplines in the past twenty years.

 These rather small numbers reflect the big gap in the current body of literature that associates brain-based learning with creativity-based disciplines, specifically in design and visual arts

 education. This infers the necessity to direct the attention of academics, researchers, and educationalists in the fields of design and arts towards brain-based learning applications, research, and pedagogy.

****

**Brain-based learning for creative disciplines** (p.3)

 BBL can help design and visual art students understand how their brains work, think, and perceive; it can also facilitate the development of creative skills with reduced frustration (El-Wakeel, 2020). Therefore, BBL provides a biologically based framework for teaching and learning in the context of design and visual arts that explains to students the concepts behind

 learning; essentially learning how to learn. This meta-concept encompasses a diverse range of strategies that reduce the amount of stress in the classrooms and achieve educational goals easier and faster. Yet, educationalists, especially in the field of design and visual arts, have not fully utilized results from neuroscience studies into BBL methodologies.

 ……

 Despite the relevance of BBL to design and arts education as creativity-inspired disciplines, there is a scarcity of published research that has made the proper connection between them.

 A very few studies were found that incorporated BBL principles into learning in the field of ‘Arts’ in general but not particularly in design and visual arts.

 P.4

 The necessity for more specialized studies and application of BBL in design and visual arts motived the authors to perform a bibliometric analysis to identify the gaps, trends, subject development, and growth in the published body of literature on BBL in design, visual arts, and creativity-based subjects. Identifying such gaps will help direct more focused and applicable future research.

**Research aim and objectives** (p.4)

 Aiming to identify the gaps in the current literature that relates brain-based learning to creative subjects such as design and visual arts.

****

**Methods**

**Data selection and method** ****(p.4)

 The topics ‘design’ and ‘creativity’ were used in the initial search and then refined by the term ‘brain-based learning,’ this allowed the retrieval of records used in this study, the following search strategy was applied: (TITLE-ABS-KEY (“Design”) OR TITLE-ABS-KEY (“Creativity”) AND (“brain-based learning”)

**Productive sources** (p.9)

 while there were only 3 sources that were directly related to design and visual arts studies and/or education, and they were not detected in the top ten results. This shows that there is a very slim opportunity for researchers to publish on platforms dedicated to this field which

 explains the extended literature gap in this area.

****

**Productive authors** (p. 9)

 On the other hand, the low impact of authors specialized in design and visual arts education raises the question of why researchers, academics, and educationalists in

 design disciplines are not directed towards liking BBL to design education despite the significance of this topic and its high relevance to enhancing design students’ knowledge acquisition and creativity.

**Analysis of author keywords**  P.12:

 Searching for keywords such as Design and Visual arts may not yield many results; however, the keyword Neuroscience was found in the same cluster as Design and Visual Arts. The occurrences were very low, but there is a direct association between these fields researchers must relate to. Creativity and Creative thinking are keywords that find somewhat relevant literature related to design disciplines.

**Thematic evaluation by topic** P.13:

 More interestingly, this unigram shows that several topics such as ‘thinking’, ‘designing’, ‘model’, ‘effect’ and ‘skills’ are not showing any relationship with the topic ‘design’, contrary to what may be assumed. The visualization also reveals that

 in 2017-2020, a few new topics have emerged such as ‘thinking’, and ‘skills’ which are strongly related to ‘mathematics’ and ‘education’. It is also worth mentioning that the topic ‘visual arts’ is missing from the unigram, meaning its frequency was not detected. The thematic evolution analysis substantiates that there is a decline in the development of BBL research relating to the topics of design, visual arts, creativity, and design education.

**Conclusion** P.18:

 This research identified the relevance of brain-based learning in the field of design and arts education as it carefully considers how the brain learns, especially in creativity-based subjects. However, the initial literature review indicated the absence of publications relating BBL pedagogical methodologies to design and visual arts education despite the associated relevance regarding these vital topics.

 ….

 A comparison of authors’ keywords with all keywords indicated that the subject approach of author keywords is direct, more precise, and relatable to the topic. However, the keywords that appeared the most in the studied publications mainly focused on learning and education in general, instructional design and collaborative learning, neuroscience, and creativity. No keywords related to ‘design’ and ‘arts’ as a topic were found. This indicates that there is a large gap in the current body of literature that relates BBL to creativity-based disciplines such as design and visual arts despite the apparent relevance between them. In addition, the thematic evolution analysis carried out in this research substantiates that there is clear regression in the development of BBL research relating to the topics of design, visual arts, creativity, and design education.

 …

 Academics, researchers, and educationalists in design and visual arts institutes are advised to direct their attention to BBL pedagogical activities and funded action research to benefit from neurosciences research on how the brain learns. This will increase students’ conscious learning, expand their imagination, improve creativity and support multiple intelligences that develop their learning experience. This will also contribute to increasing the number of

 studies aiming at improving BBL methods to keep pace with the global challenges in education that we are currently facing.
